# Thermal Conductivity and Microstructure of Novel Flaxseed-Gum-Filled Epoxy Resin Biocomposite: Analytical Models and X-ray Computed Tomography

**DOI:** 10.3390/ma16186318

**Published:** 2023-09-20

**Authors:** Mohammed Zaidi, Dominique Baillis, Naim Naouar, Michael Depriester, François Delattre

**Affiliations:** 1 Unité de Chimie Environnementale et Interactions sur le Vivant (UR 4492, UCEIV), SFR Condorcet FR CNRS 3417, Littoral Côte d’Opale University, 145 Avenue Maurice Schumann, 59140 Dunkerque, France; 2 LaMCoS, INSA-Lyon, CNRS UMR 5259, Université de Lyon, 69621 Villeurbanne, France; 3 Unité de Dynamique et Structure des Matériaux Moléculaires (UR 4476, UDSMM), Littoral Côte d’Opale University, 145 Avenue Maurice Schumann, 59140 Dunkerque, France

**Keywords:** flax fiber, flaxseed gum, biocomposite material, thermal conductivity, analytical models, X-ray computed tomography

## Abstract

The growing awareness of the environment and sustainable development has prompted the search for solutions involving the development of bio-based composite materials for insulating applications, offering an alternative to traditional synthetic materials such as glass- and carbon-reinforced composites. In this study, we investigate the thermal and microstructural properties of new biocomposite insulating materials derived from flaxseed-gum-filled epoxy, with and without the inclusion of reinforced flax fibers. A theoretical approach is proposed to estimate the thermal conductivity, while the composite’s microstructure is characterized using X-ray Computed Tomography and image analysis. The local thermal conductivity of the flax fibers and the flaxseed gum matrix is identified by using effective thermal conductivity measurements and analytical models. This study provides valuable insight into the thermal behavior of these biocomposites with varying compositions of flaxseed gum and epoxy resin. The results obtained could not only contribute to a better understanding the thermal properties of these materials but are also of significant interest for advanced numerical modeling applications.

## 1. Introduction

Today, increasing global consumption is accelerating the scarcity of resources, and developing alternatives poses a major challenge, particularly in the context of climate change and the replacement of fossil fuels [1]. Consequently, it is time to promote the use of renewable resources whose agro-sourced origin would enable the integration of a circular economy with a regional and local dimension. The use of agro-resources, in particular agricultural residues, holds great interest for the development of biocomposite materials, offering both environmental benefits and competitive production costs [2]. Natural biocomposites have great potential in a wide range of applications, including in the automotive industry, packaging, and household goods [3,4]. Indeed, they can serve as cost-effective materials while offering a wide range of structural properties [5]. Fiber-reinforced biocomposites, especially those derived from flax, are emerging as promising substitutes for synthetic fibers in polymeric composites. They have attracted interest due to their ecological attributes, cost-effective production, and mechanical characteristics. Several studies have taken advantage of them in various applications, particularly the automotive industry and building insulation [6,7,8].

Due to their interesting mechanical and thermal properties, the characteristics of flax-fiber-based biocomposites was investigated by various researchers [9,10,11,12,13]. In [14], a biocomposite was developed using low-cost raw materials resulting from the flax industry. Non-woven flax fibers were chosen as a reinforcement, while mucilage polysaccharides, extracted from flaxseeds, served as a matrix. [15] investigated the possibility of using flax stems as reinforcement in a polylactic acid (PLA) matrix to produce a lightweight, fully bio-based composite with improved mechanical properties. Additionally, [16] provided an overview of flax fibers, the methods used to process these fibers, and the composites developed using different types of matrices. This overview aims to provide a succinct and fundamental resource for future research into flax-based composites.

Recently, [17,18] developed a new biocomposite-based flax fiber and flaxseed gum extracted from flax seeds as a matrix. This combination has exhibited interesting thermal and mechanical properties. An extensive thermal characterization of these biocomposite materials was conducted by considering various ratios of flaxseed gum, epoxy resin, and flax fibers. However, measuring the intrinsic thermal conductivity of individual components such as short flax fibers and flaxseed gum within the composite has proven to be challenging. Obtaining these thermal properties is essential, especially for modeling the thermal conductivity of the composites while varying their composition.

In the present study, a combined experimental and theoretical approach was proposed to estimate the unknown thermal properties of these insulating biocomposites. This approach is based on using analytical models that require the microstructural characteristics for each component, including porosity, fiber volume fraction, and matrix volume fraction [19,20].

In the literature, microstructure characterization of the biocomposites can be conducted using X-ray Computed Tomography (X-ray CT) imaging and image analysis. This technique enables the assessment of both quantitative and qualitative information in three dimensions [21,22]. It was used to quantify the volume fraction, dimensions, and orientation distribution of jute [23] and carbon fiber in composites [24]. To analyze porosity, [25] investigated the size distribution and morphology of pore networks in carbon fiber/epoxy composites using X-ray CT, while [26] analyzed the porosity and microstructure of natural coir fibers for their potential as reinforcements in composites. In the context of improving braided composites, [12] studied the effect of voids in bio-based composites formed from natural fibers and bio-resin. Additionally, image processing techniques were used to calculate the size, pore size distribution, and shape of polycaprolactone-based biocomposites [13].

The current study aimed to predict the effective thermal conductivity of a novel flax-based biocomposite insulating material. The porosity and volume fraction of each component were thoroughly obtained from image analysis. Such data are required for thermal conductivity calculations. The originality of this study lies in the combined experimental and analytical approach used to estimate the intrinsic conductivity of each component of the material. Indeed, it is particularly difficult to measure them directly using typical experimental devices. In addition, this study also aims to explore the potential applications of these biocomposite as insulating materials in various applications.

## 2. Materials and Methods

**Figure 1:**
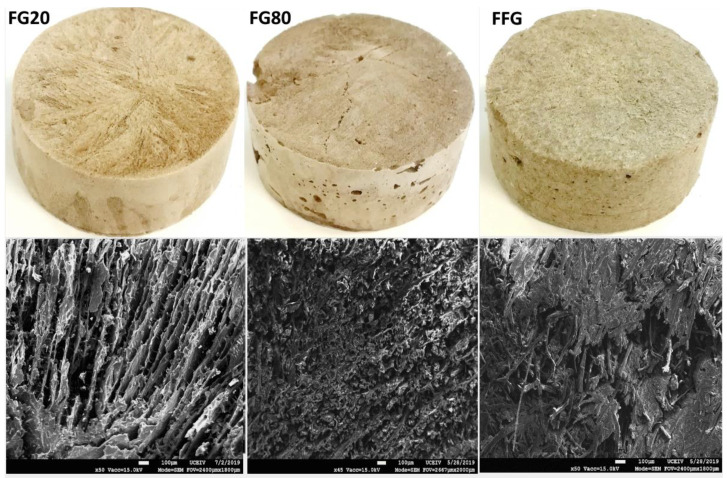
Pictures and SEM images of the cross-section morphology of the three composites: FG20, FG80, and FFG.

Flaxseed gum polysaccharide (FG) used as a matrix for the composites was extracted according to the procedure described in [27]. The extraction process involved mixing 10% flax seeds with tap water and soaking for 1 h at 40 °C for 6 h with a stirring speed of 400 rpm. After filtering the seeds, the flax gum was precipitated with ethanol [8]. The epoxy resin (Epolam 2020) and its hardener were purchased from Sigma Aldrich, St. Louis, MO, USA. The flax fibers used for reinforcing composites were purchased from Van Robaeys Frère (Guillem, France) and were 1 mm in length. Biocomposite samples were prepared as follows: flaxseed gum (mucilage) was mixed with 160 mL of water and left to stand for 12 h. Then, epoxy resin and fibers (if used) were added to the polysaccharide solution to reach a mixture containing 20% organic matter. This mixture was poured into a silicone mold (44 mm in diameter and 2 mm in height) and frozen at −80 °C. Subsequently, the samples were subjected to freeze drying for 72 h at 2 mbar (primary drying), followed by an additional 24 h at 0.002 mbar (secondary drying). Once the freeze-drying process was completed, the samples were baked in an oven at 80 °C for 5 h. Finally, the samples were polished to create cylindrical samples that were 20 mm in height with parallel top and bottom surfaces. Figure 1 shows the pictures of three samples and the images of their cross-section structure obtained with scanning electron microscopy (SEM).

### 2.1. Description of Materials

Table 1 presents the composition, density, and measured thermal conductivity of the fabricated samples. A total of six samples were analyzed, with different mass ratio of flaxseed gum, epoxy resin, and flax fibers. The FG100, FG80, and FG20 samples do not contain flax fibers and are composed of 100%, 80%, and 20% by mass ratio of flaxseed gum, respectively, with the remaining proportion being epoxy resin. The fibrous flaxseed gum composites (FFG) contain a mixture of flaxseed gum, epoxy resin, and flax fibers. A dense sample of epoxy resin was also analyzed. The sample, referred to as “chopped fibers,” consists of compressed panels entirely made of random flax fibers measuring 1 mm in length. 

Bulk density measurements (Table 1) were performed using an Electronic Digital LCD Gauge Stainless Nonius Caliper Micrometer 150 mm 6-inch MIS and a VWR LP 3102 balance. Composite samples were polished with a Struers Tegrapol 21 polisher and 80 g/cm^2^ sandpaper to obtain parallel surfaces and a height of 20 ± 0.5 mm. For this, the volume of the samples was measured with calipers (4 samples per test) and weighed to an accuracy of 0.01 g.

Thermal conductivity measurements of materials were conducted using a C-Therm Technologies TCi Analyzer. The system is based on a modified transient plane source (MTPS) technique. It is important to note that all measurements were performed at ambient temperature. The experimental procedure conforms to ASTM D7984 [28].

### 2.2. Microstructural Characterization of Samples

The samples underwent characterization using X-ray CT and image analysis to analyze their microstructure and quantify the porosity and volume fraction of each component. The X-ray CT scans were conducted at INSA Lyon, France, using a laboratory X-ray scanner. The data acquisition system recorded a total of 1120 projections, evenly distributed over a 360° rotation along the vertical axis of the sample. To obtain the 3D reconstructions, the recorded projections were processed using Xact software (http://xactsoftware.co.in/ accessed on 5 September 2023).

To optimize both the quality of the images and the number of scans, the samples were scanned in smaller representative elementary volumes (REVs) of approximately 1 cm^3^. For each sample, a total of four REVs scans were randomly performed, with 8 µm in voxel size. It is important to note that during the image analysis, pores, and flax fibers in contact with the edges of the REV were excluded from the calculation.

**Figure 2:**
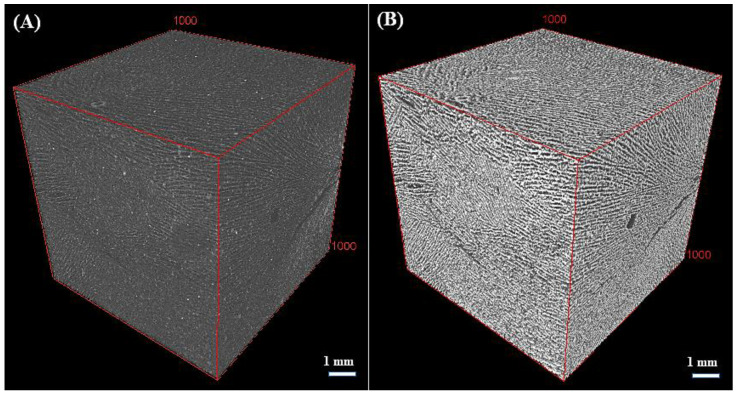
Example of FG20 sample segmentation using LABKIT plug-in. (**A**) Original X-ray CT image, and (**B**) binary image.

Segmentation of the images was conducted using the LABKIT plug-in [29], which is an integrated feature within the ImageJ software [30]. An example of the quality of segmentation is shown in Figure 2, where black voxels represent void spaces, and grey voxels correspond to the solid phase within the sample.

To determine the volume fraction of each component within the composite, we divided the volume of the segmented component by the total volume of the REV. This process was repeated for each REV, and the average volume fraction was then calculated. 

### 2.3. Analytical Approach for Estimating Thermal Conductivity

This section focuses on the proposed theoretical approach based on three steps. Firstly, analytical models allow the estimation of the effective thermal conductivity of fibrous composites, including chopped flax fibers (in air). Then, analytical models are used to determine the effective thermal conductivity of the porous matrix of composites. Finally, mixing laws are used to predict the local thermal conductivity (non-porous) of the matrix, *λ_FG_*_(*s*)_, with a given FG100/epoxy volume fraction ratio.

#### 2.3.1. Thermal Conductivity of the Fibrous Media

The analytical model of Schuetz and Glickman [31] allows for the prediction of the thermal conductivity of fibrous media. This model was previously used in similar types of bio-based composites containing juncus maritimus fiber media [32]. The effective thermal conductivity is determined using the following expression:

(1) $$\lambda_{eff}=\lambda_{FG}({1-\nu }_{Fb})+\nu_{Fb}\left(\frac{2-f_{s}}{3}\right)\lambda_{Fb(s)}$$

with ${1-\nu }_{Fb}=\varphi +\nu_{FG(s)}$,
where,
*λ_eff_*: is the effective thermal conductivity of the fibrous composite.
*λ_FG_*: is the effective thermal conductivity of the porous flaxseed gum (FG) matrix.
*λ_Fb_*_(s)_: is the local thermal conductivity of the flax fibers (s = solid).
*ν_Fb_*: is the volume fraction of the flax fibers.
*φ*: is the porosity.
*f_s_*: is a morphological parameter (*fs* = 1 for fibers).

It can be noted that this model can be used in the case of fibrous composites in air [26].

In this case, $\lambda_{FG}\;$is replaced by *λ_g_*:


(2) $$\lambda_{eff}=\lambda_{g}({1-\nu }_{Fb})+\nu_{Fb}\left(\frac{2-f_{s}}{3}\right)\lambda_{Fb(s)}$$

with ${1-\nu }_{Fb}=\varphi$.
where,
*λ_g_*: is the thermal conductivity of the air.

#### 2.3.2. Thermal Conductivity of the Porous Flaxseed Gum Matrix

As presented in the materials and methods section, the non-fibrous composites are made from flaxseed-gum-filled epoxy resin. To determine the conductivity of the non-porous phase in the matrix *λ_FG_*_(*s*)_, three analytical models were selected: Russell, Maxwell, and Bruggeman [33,34]. These models were chosen based on their capability for estimating thermal conductivity for different shapes of pores within the composite material. 

Russell proposed a mathematical model (3) to predict the thermal conductivity in composites composed of cubic cells arranged in a row, i.e., series and parallel arrangements:

(3) $$\lambda_{FG}=\frac{\lambda_{FG(s)}\left[\lambda_{FG(s)}+\varphi^{\frac{2}{3}}(\lambda_{g}-\lambda_{FG(s)})\right]}{\lambda_{FG(s)}+(\lambda_{g}-\lambda_{FG(s)})(\varphi^{\frac{2}{3}}-\varphi )}$$

where the components are as follows:

*λ_FG_*: is the effective thermal conductivity of the porous flaxseed gum matrix.
*λ_FG_*_(*s*)_: is the local thermal conductivity of the non-porous flaxseed gum matrix.
*φ*: is the porosity of the flaxseed gum matrix.

The Maxwell model, also known as the Maxwell–Eucken model, is an effective medium approximation used to estimate the thermal conductivity of porous materials. This model (4) assumes that the fluid phase is composed of randomly dispersed spheres [33]. The equation of the effective thermal conductivity of a porous matrix can be expressed as follows:

(4) $$\lambda_{FG}=\lambda_{FG(s)}\frac{\lambda_{g}+2\lambda_{FG(s)}+2\varphi (\lambda_{g}-\lambda_{FG(s)})}{\lambda_{g}+2\lambda_{FG(s)}-\varphi (\lambda_{g}-\lambda_{FG(s)})}$$

The Bruggeman model, also referred to as the Bruggeman homogenization model, is used to predict the effective thermal conductivity of porous materials that contain spherical or cylindrical inclusions [35]. In this study, the pores can be assumed to be nearly spherical, and the expressions for the effective thermal conductivity can be expressed as follows (5):

(5) $$\;\;\;\lambda_{FG}=\frac{\lambda_{FG(s)}\left[1-(1-\frac{\lambda_{g}}{\lambda_{FG(s)}})\frac{2}{3}\varphi \delta \right]}{\left[1+(\delta -1)\varphi \right]}$$

where *δ* is determined from the Equation (6) for spherical particles:

(6) $$\delta =\frac{3\lambda_{FG(s)}}{2\lambda_{FG(s)}+\lambda_{g}}$$

#### 2.3.3. Local Thermal Conductivity of the Flaxseed Gum Matrix

To estimate the local thermal conductivity of the non-porous matrix, *λ_FG_*_(*s*)_, with a given volume fraction ratio of FG and epoxy resin, the analytical models require determination of the intrinsic thermal conductivity of FG and epoxy resin in the matrix. The thermal conductivity of the solid phase can be calculated using the mixing law of series (7) or parallel (8) mixing laws:

(7) $$\frac{1}{\lambda_{FG(s)}}=\frac{X}{\lambda_{FG100(s)}}+\frac{\left(1-X\right)}{\lambda_{Epoxy(s)}}$$

(8) $$\lambda_{FG(s)}=X\lambda_{FG100(s)}+(1-X)\lambda_{Epoxy(s)}$$

where,

*X*: is the volume fraction of the pure flaxseed gum (FG100) in the solid matrix.
*λ_FG_*_100(*s*)_: is the intrinsic thermal conductivity of the non-porous FG100.
*λ_Epoxy_*_(*s*)_: is the intrinsic thermal conductivity of the non-porous epoxy resin.

## 3. Results and Discussion

### 3.1. Microstructure Analysis

**Figure 3:**
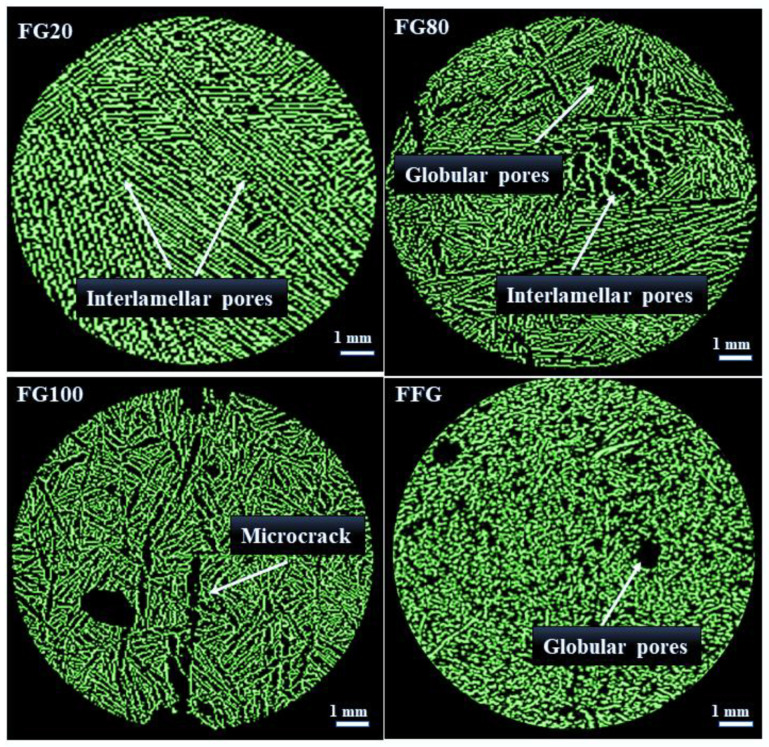
2D visualization of the sample’s microstructure and pores shape.

Figure 3 shows the microstructure of the samples obtained from X-ray CT images with a voxel size of 8 µm. The porosity network within flaxseed-gum-filled epoxy composites, encompassing different ratios of flaxseed gum/epoxy, highlights the presence of three distinct types of pores: (i) inter-lamellar pores separating two adjacent lamellae, with an average distance ranging from 2 to 300 µm; (ii) spherical and sub-spherical globular pores resulting from air trapped in the matrix during the fabrication process, which have an average maximum diameter of 1500 µm; and (iii) microcracks that developed due to the thermal stresses during the freezing process, with an orientation identical to that of the inter-lamellar pores. 

The parallel lamellar alignment structure showed in samples FG20, FG802 and FG100 is a result of the extraction of lyophilized ice crystals in the vertical direction of the specimens. This similar lamellar structure was observed in previous studies involving graphene/epoxy and ceramic composites [36,37]. The architecture of FG20 displays a well-organized arrangement of lamellae, while FG80 and FG100 show some defects and microcracks. Reducing the epoxy content from 80% to 20% leads to a trend towards a less structured microstructure. In the fibrous composite (FFG), the incorporation of flax fibers into the flaxseed gum matrix resulted in substantial alterations to its microstructure. The images also revealed more spherical pores compared to the FG20 and FG80 samples, indicating good compatibility between the fibers and the matrix. Additionally, the absence of cracks and deformations in the fibrous composite suggests good structural cohesion.

Table 2 summarizes the porosity and volume fraction of the flaxseed-gum-filled epoxy and fibers in the samples obtained from the segmentation method. The porosity values range from ~61% ± 3.0 for FG20 to ~68% ± 2.4 for FG80 and FG100. This increase in porosity was attributed to alterations in the microstructure, such as the opening of inter-lamellar pores (Figure 3), as well as the development of some microcracks in FG80 and FG100 due to the increase in the volume fraction of the flaxseed gum in the matrix. These microcracks create additional pathways for fluid flow within the matrix, leading to an overall higher porosity. These results also highlight the impact of the mucilage/epoxy ratio on the porosity within the matrix. In addition, the panel made from chopped fibers (in air) has a higher porosity (76% ± 1.0) due to the absence of a fiber-binding component.

### 3.2. Thermal Conductivity Estimation

To determine the effective thermal conductivity of heterogeneous materials using analytical models, it is necessary to know the thermal conductivity of each component constituting the composites. As these are porous materials, it is essential to determine the conductivity of the non-porous phase of the matrix, as well as that of the flax fibers. Furthermore, as the matrix itself is made up of pure flaxseed gum (FG100) and epoxy resin, it is crucial to identify the conductivity of each of these components separately. The following sections discuss in detail the method used to determine the thermal conductivity of each component of the composite, using analytical models and mixing laws.

#### 3.2.1. Thermal Conductivity of the Non-Porous Flaxseed-Gum-Filled Epoxy Matrix

**Figure 4:**
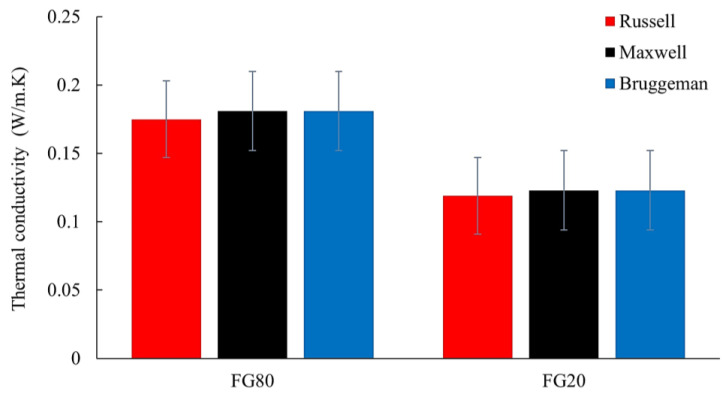
Estimated local thermal conductivity of the non-porous flaxseed gum matrix.

As shown in Figure 3, the segmented images of the non-fibrous composites revealed two clearly identifiable phases: a continuous solid phase composed of flaxseed-gum-filled epoxy and a dispersed phase corresponding to the pores. However, since flaxseed gum and epoxy resin have a close attenuation coefficient, the images showed only one component, making it difficult to distinguish between the two components. Thus, both flaxseed gum and epoxy were considered as a one solid component. To determine the local thermal conductivity of this non-porous phase, the three analytical models Russell (3), Maxwell (4), and Bruggeman (5) (Cf. Section 3.2), based on different pore shapes, were used. As shown in Figure 4, the histograms present the variation in the local thermal conductivity of the two heterogeneous samples FG20 and FG80. These results indicate minimal discrepancies among the conductivities obtained from the three models. This suggests that the shape of the pores has limited influence on the thermal conductivity variation. The porosity has a more significant impact on conductivity than pore shape. Thus, in the next section, the usual Maxwell model is retained for its compatibility with the spherical shape of the pores present in the matrix, compared with the other tested models. The conductivity of the non-porous samples FG80 and FG20 are 0.181 and 0.123 W·m^−1^·K^−1^, respectively.

#### 3.2.2. Local Thermal Conductivity of Chopped Flax Fibers

**Figure 5:**
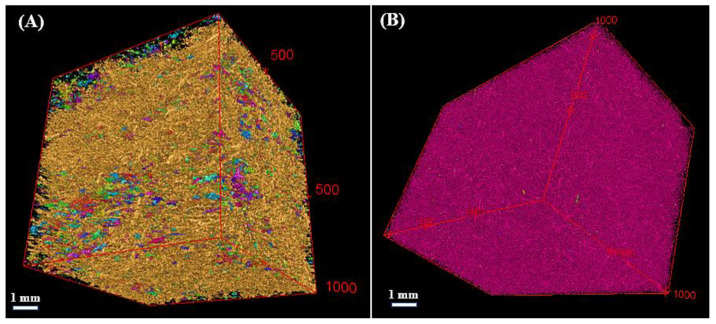
X-ray CT images of the segmented flax fibers from (**A**) the fibrous composite (FFG) and (**B**) the panel’s fibers (in air).

The thermal conductivity measurements obtained from panels of chopped fibers (1 mm in length) were used to estimate the intrinsic thermal conductivity of individual flax fibers (without porosity). The analytical model of Schuetz and Glickman (2) was applied to a panel containing randomly oriented fibers in air (Figure 5B). This model considers the thermal conductivity of the solid fibers *λ_Fb_*_(*s*)_, the porosity, and the morphological parameter *fs* of the solid phase. Considering the experimental thermal conductivity (0.048 W·m^−1^·K^−1^) and the porosity (76% ± 1.0) obtained from X-ray CT images, the local thermal conductivity of the fibers, *λ_Fb_*_(*s*),_was determined using the following expression:

(9) $$\lambda_{Fb(s)}=\frac{3}{2-fs}\frac{\lambda_{Fb}-\varphi \lambda_{g}}{1-\varphi }=0.35(W\cdot {m^{-1}\cdot K}^{-1})$$

where,

*λ_Fb_*: is the effective thermal conductivity of the flax fibers (in air).
*λ_g_*: is the thermal conductivity of the air (*λ_g_* = 0.026 W·m^−1^·K^−1^).

In the literature and to our knowledge, there are no previous studies available in the literature that specifically provide the local thermal conductivity of randomly oriented short flax fibers. However, for well-oriented flax fibers, a study conducted by [38] provided results of experimental measurements of the thermal conductivity of flax fibers. In the longitudinal direction, the thermal conductivity was 1.232 (W·m^−1^·K^−1^), while in the transverse direction, it was 0.17 (W·m^−1^·K^−1^). These measurements diverge from our results because we used shorter and randomly oriented fibers, compared to their well-oriented fibers.

Note that in the Section 3.2.4, a second method for determining fiber conductivity based on the experimental conductivity of the fibrous composite will be proposed. The results obtained from both methods will then be compared and discussed.

#### 3.2.3. Intrinsic Thermal Conductivity of the Pure Flaxseed Gum (FG100) and Epoxy Resin

To estimate the matrix’s thermal conductivity for a given flaxseed gum/epoxy ratio, it is necessary to identify the intrinsic conductivity of the pure flax gum (FG100) and the epoxy resin present in the matrix. For this purpose, the experimental conductivities of the three samples, FG100, FG80, and the epoxy resin, were used. The effective conductivity of their solid phase (non-porous) was deduced from Maxwell’s model by knowing the porosity (Table 2). Then, knowing the volume fractions of the pure flaxseed gum (FG100) and epoxy in the matrix, their local conductivity (FG100 and epoxy) was deduced using the series and parallel mixing laws (Equations (7) and (8)).

The volume fraction of epoxy resin *ν_Epoxy_*_(*s*)_ in each sample was determined using Equation (10) [32], where *m_Epoxy_*_(*s*)_ represents the mass fraction of the epoxy and *ρ_matrix_*_(*s*)_*/ρ_Epoxy_*_(*s*)_ is the ratio of the density of the matrix to that of the epoxy (Table 1).

(10) $$\nu_{Epoxy(s)}=m_{Epoxy\left(s\right)}\frac{\rho_{matrix(s)}}{\rho_{Epoxy(s)}}$$

where,

$$\rho_{matrix(s)}=\frac{\rho_{matrix}}{1-\varphi }$$

with


ν_FG(s)_ = 1 − ν_Epoxy(s)_

**Figure 6:**
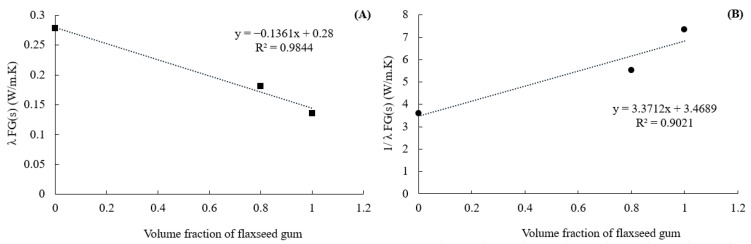
The linear trend curves obtained from (**A**) parallel (Equation (11)) and (**B**) series mixing laws (Equation (12)).

By knowing the volume fractions (Table 3) and the conductivity of the non-porous fraction of the three samples, FG100, FG80, and the epoxy resin (Table 1), the thermal conductivity of FG100 and epoxy resin can be obtained from mixing laws. From the linear trend curves of Equations (11) and (12), the slope and y-intercept can be deduced. This yields *λ_FG100_* and *λ_Epoxy_* (Figure 6).

(11) $$\frac{1}{\lambda_{FG(s)}}=X\left(\frac{1}{\lambda_{FG100\left(s\right)}}-\frac{1}{\lambda_{Epoxy\left(s\right)}}\right)+\frac{1}{\lambda_{Epoxy(s)}}$$

(12) $$\lambda_{FG(s)}=X{(\lambda }_{FG100(s)}-\lambda_{Epoxy\left(s\right)})+\lambda_{FG100(s)}$$

For FG100 sample, the thermal conductivities (*λ_FG100_*) were around 0.056 and 0.057 (W·m^−1^·K^−1^) for both parallel and series models, respectively. Thus, an average value of 0.0565 W·m^−1^·K^−1^ can be considered for FG100. The thermal conductivity of epoxy resin is around 0.28 and 0.288 (W·m^−1^·K^−1^) for parallel and series models, respectively. Thus, a mean value of 0.284 W·m^−1^·K^−1^ can be considered for epoxy. It can be noted that in the literature, the thermal conductivity of epoxy varies between 0.18 and 0.26 (W·m^−1^·K^−1^) [39,40]. These values are of the same order of magnitude as that obtained and tend to show the validity of the approach.

#### 3.2.4. Local Thermal Conductivity of Flax Fibers Based on the Effective Conductivity of the Fibrous Composite

In Section 3.2.2, the local thermal conductivity of the flax fibers was estimated from the experimental conductivity of the chopped fibers in air. An alternative method is also proposed for determining the local conductivity of fibers from the fibrous composite (FFG) (Figure 5A). 

Given the known thermal conductivities of FG100 and epoxy resin, and their respective volume fractions (Table 3), the thermal conductivity of the non-porous FFG matrix was deduced: *λ_FG_*_(*s*)_ was around 0.180 W·m^−1^·K^−1^. Considering that the porosity of FFG is 65% (Table 2) and by applying Maxwell’s model, the estimated thermal conductivity of the porous matrix, *λ_FG_*, was 0.069 W·m^−1^·K^−1^.

Using the fiber volume fraction obtained through image analysis, the conductivity of the porous matrix (0.069 W·m^−1^·K^−1^) and the experimental effective conductivity of the FFG (*λ_eff_*), the local conductivity of the fibers, *λ_Fb_*_(*s*)_, was deduced from Equation (13). The obtained value was approximately 0.123 (W·m^−1^·K^−1^).

(13) $$\lambda_{Fb(s)}=\frac{3}{2-fs}\frac{\lambda_{eff}-\left({1-\nu }_{Fb}\right)\lambda_{FG}\;\;}{\nu_{Fb}}=0.123(W\cdot {m^{-1}\cdot K}^{-1})$$

Note that this value is lower than the *λ_Fb_*_(*s*)_ = 0.35 (W·m^−1^·K^−1^) obtained in Section 3.2.2. In [32,41], this overestimation of the thermal conductivity of fibers in air was explained by the potential contribution of radiation inside highly porous Juncus maritimus fibers. Accordingly, the obtained value (0.123 W·m^−1^·K^−1^) is close to the average thermal conductivity of treated flax fibers 0.1187 (W·m^−1^·K^−1^), obtained by [19].

#### 3.2.5. Estimating the Thermal Conductivity of the Porous Matrix for Different Volume Fractions of Flaxseed Gum

**Figure 7:**
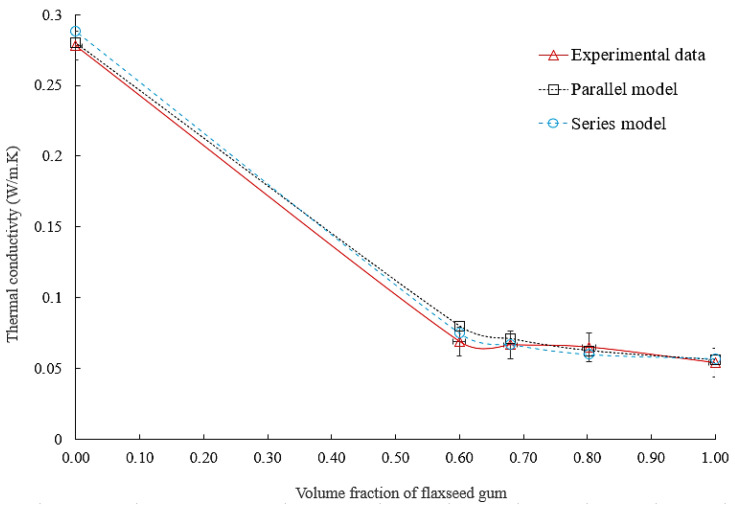
Comparison of flaxseed gum matrix thermal conductivity to experimental data.

Figure 7 illustrates the evolution of flaxseed gum conductivity as a function of FG100 volume ratio. The conductivities of FG100 and epoxy were obtained by fitting linear trend curves using the two mixing laws in series and parallel, as discussed in Section 3.2.3. This curve represents all experimental and theoretical data points concerning matrix thermal conductivity in both fibrous and non-fibrous composites. A good agreement between experimental and theoretical results was observed. Since the parallel and series mixing laws yield values close to each other, the average value between the two models could be considered as a reliable estimation of the thermal conductivity of the composite matrix.

## 4. Conclusions

The present study aims to estimate the thermal conductivity of newly developed insulating composites made from flax fibers and a flaxseed-gum-filled epoxy matrix. An analytical approach was applied to estimate the local thermal conductivity of the matrix and fibers using analytical models. In addition, the microstructure of the composites was characterized using X-ray CT and image analysis.

The composite’s microstructure shows a lamellar structure with three types of pores: inter-lamellar, spherical and microcracks. The parallel alignment of the lamellae is due to the freeze-drying process. The lamellar structure of the flaxseed gum matrix, containing 80% epoxy (FG20), appears more organized and uniform than FG20 and FG100. The incorporation of reinforced fibers in the matrix (FFG sample) results in a structural modification that reinforces the composite’s cohesion.

To overcome the lack of experimental data on the local thermal conductivity of the solid (non-porous) phase in composites, analytical models based on the two-phase continuous/discontinuous principle were used in this study. The results revealed that the shape of the pores has a negligible effect on the conductivity variation, while the porosity significantly influences the thermal conductivity of the composite material.

Since the matrix is made up of two homogeneous components, FG100 and epoxy resin, determining the conductivity of each component is crucial to assessing the overall conductivity of the matrix. Consequently, the average conductivity of pure flaxseed gum FG100 (0.145 W·m^−1^·K^−1^) and pure epoxy resin (0.284 W·m^−1^·K^−1^) was estimated using both parallel and series mixing laws.

The local thermal conductivity of flax fibers was determined from chopped flax fibers (in air) 1 mm long. Glickman’s analytical model was used for this calculation, revealing a thermal conductivity of 0.35 W·m^−1^·K^−1^. However, this value may be overestimated due to the radiation contribution. An alternative estimation was therefore proposed, based on the fibrous composite (FFG). As expected, the fiber’s thermal conductivity obtained was around 0.123 W·m^−1^·K^−1^. This value is close to the average thermal conductivity of the pure flaxseed gum, FG100 (0.145 W·m^−1^·K^−1^).

Obtaining the thermal conductivity of this composite material can be challenging, as it is difficult to directly measure the intrinsic thermal conductivity of each (non-porous) component using conventional experimental devices. However, determining the intrinsic conductivity of each component of the composite material could provide the basis for more advanced numerical modeling of the composite material in the future.

Finally, the thermal properties of these materials highlight their ability to provide effective thermal insulation, allowing the development of lightweight biocomposites capable of competing with synthetic foams commonly used in the automotive and building insulation fields. The renewable nature, natural origin, and biodegradability of these materials make them attractive for the development of competitive products in these sectors.

## Figures and Tables

**Table 1:** Mass fraction and thermo-physical properties of composite samples.

Sample	m_FG_ (%)	m_Fibers_ (%)	m_Epoxy_ (%)	Bulk Density (g/cm^3^)	Thermal Conductivity (W·m^−1^·K^−1^)
FG100	100.0	0.0	0.0	228.9 ± 9.3	0.054 ± 0.001
FG80	80.0	0.0	20.0	0.231 ± 8.2	0.065 ± 0.001
FG20	20.0	0.0	80.0	0.219 ± 3.0	0.057 ± 0.001
FFG	12.0	48.0	40.0	0.194 ± 5.3	0.064 ± 0.001
Epoxy resin (dense)	0.0	0.0	100	1.1	0.782 ± 0.001
Chopped fibers (1 mm in length)	-	100.00	-	0.108	0.048 ± 0.001

**Table 2:** Porosity and volume fraction of the flaxseed-gum-filled epoxy and fibers in the samples.

Sample	*ν_FG_* (%)	*ν_fibers_* (%)	Porosity (%)
FG100	32.0	-	68.0 ± 2.4
FG80	32.0	-	68.0 ± 2.5
FG20	39.0	-	61.0 ± 3.0
FFG	19.0	16.0	65.0 ± 1.1
Chopped fibers (1 mm)	-	24.0	76.0 ± 1.0

**Table 3:** Volume fraction of FG100 and epoxy resin in the matrix.

Sample	ν_FG100_ (%)	ν_Epoxy_ (%)
FG100	100.00	0.00
FG80	80.30	19.70
FG20	59.20	40.80
FFG	35.00	65.00
Epoxy resin	0.00	100.00

## Data Availability

No data available.
